# WT1 regulates angiogenesis in Ewing Sarcoma

**DOI:** 10.18632/oncotarget.1610

**Published:** 2014-02-03

**Authors:** Varalakshmi Katuri, Stephanie Gerber, Xiaofei Qiu, Gregory McCarty, Seth D. Goldstein, Hans Hammers, Elizabeth Montgomery, Allen R. Chen, David M. Loeb

**Affiliations:** ^1^ Division of Pediatric Oncology, Sidney Kimmel Comprehensive Cancer Center, Johns Hopkins University, Baltimore, MD; ^2^ Division of Genitourinary Oncology, Department of Oncology, Sidney Kimmel Comprehensive Cancer Center, Johns Hopkins University, Baltimore, MD; ^3^ Department of Pathology, Johns Hopkins University, Baltimore, MD

**Keywords:** tumor angiogenesis, transcriptional regulation, tissue microarray, vascularity, alternative splicing

## Abstract

Angiogenesis is required for tumor growth. WT1, a protein that affects both mRNA transcription and splicing, has recently been shown to regulate expression of vascular endothelial growth factor (VEGF), one of the major mediators of angiogenesis. In the present study, we tested the hypothesis that WT1 is a key regulator of tumor angiogenesis in Ewing sarcoma. We expressed exogenous WT1 in the WT1-null Ewing sarcoma cell line, SK-ES-1, and we suppressed WT1 expression using shRNA in the WT1-positive Ewing sarcoma cell line, MHH-ES. Suppression of WT1 in MHH-ES cells impairs angiogenesis, while expression of WT1 in SK-ES-1 cells causes increased angiogenesis. Different WT1 isoforms result in vessels with distinct morphologies, and this correlates with preferential upregulation of particular VEGF isoforms. WT1-expressing tumors show increased expression of pro-angiogenic molecules such as VEGF, MMP9, Ang-1, and Tie-2, supporting the hypothesis that WT1 is a global regulator of angiogenesis. We also demonstrate that WT1 regulates the expression of a panel of pro-angiogenic molecules in Ewing sarcoma cell lines. Finally, we found that WT1 expression is correlated with VEGF expression, MMP9 expression, and microvessel density in samples of primary Ewing sarcoma. Thus, our results demonstrate that WT1 expression directly regulates tumor angiogenesis by controlling the expression of a panel of pro-angiogenic genes.

## INTRODUCTION

WT1 is a transcriptional regulatory protein that is overexpressed in a wide variety of tumor types, including leukemia, breast cancer, and sarcomas [1-4]. In osteosarcoma, soft tissue sarcomas, and breast cancer patients, WT1 expression confers a poor prognosis [5-7]. Although the role of WT1 in sarcoma biology remains unclear, work from our laboratory, and from others, has implicated WT1 in the regulation of angiogenesis. WT1 is upregulated by hypoxia in endothelial cells in a coronary artery ligation model of myocardial infarction [8], and WT1 expression has also been demonstrated in tumor endothelial cells [9]. Our laboratory demonstrated that WT1 is upregulated by hypoxia in Ewing sarcoma cells *in vitro*, and that in these cells, WT1 is a direct positive regulator of vascular endothelial growth factor (VEGF) expression [10]. We further demonstrated that blocking the hypoxia-mediated upregulation of WT1 blunts the hypoxia-mediated upregulation of VEGF in these cells, supporting a functional role of WT1 in the response to hypoxia.

WT1 is a C_2_H_2_ zinc finger transcription factor. Multiple WT1 isoforms can be generated from two independent alternative splicing events [11] and the use of multiple translation initiation sites [12, 13]. The first alternative splice includes or excludes 17 amino acids encoded by exon 5, and the second alternative splice, referred to as the KTS insert, determines the inclusion of three amino acids (Lys, Thr, and Ser) between exons 9 and 10, which encode the 3^rd^ and 4^th^ zinc fingers. Individual WT1 isoforms are named based on the presence or absence of exon 5 and presence or absence of the KTS insert, such that the isoform lacking both exon 5 and the KTS insert is referred to as WT1 (-Ex5/-KTS). An alternate nomenclature designates the major isoforms as A, B, C, and D, and we will refer to WT1 (-Ex5/-KTS) as isoform “A” and WT1 (+Ex5/+KTS) as isoform “D”.

Interestingly, WT1 has been implicated in both transcriptional and post-transcriptional regulation of gene expression. All 4 WT1 isoforms bind DNA, although the binding site for isoforms lacking the KTS insert (including isoform A) is better defined [14-16] than the binding site for isoforms containing the KTS insert (such as isoform D) [17, 18]. WT1 has also been implicated in mRNA splicing, especially isoforms containing the KTS insert [19]. VEGF appears to be a key WT1 target gene, since WT1 directly regulates VEGF transcription and indirectly regulates splicing. Our laboratory showed that WT1 binds to the VEGF promoter *in vivo* and activates transcription, and Amin et al. demonstrated that WT1 represses the splice factor kinase SRPK1, whose target, SRSF1, directly regulates the splicing of VEGF, specifically the utilization of either exon 8a or exon 8b in the mature mRNA [10, 20].

Based on our observation that WT1 can upregulate VEGF in Ewing sarcoma cell lines, we tested the hypothesis that WT1 regulates tumor angiogenesis in Ewing sarcoma xenografts and in primary Ewing sarcoma tumors. We confirmed that WT1 expression positively regulates angiogenesis in Ewing sarcoma xenografts, and found that WT1 modulates VEGF isoform expression as well. In addition to VEGF, we also demonstrate that WT1 regulates the expression of a number of other target genes that influence angiogenesis, including angiopoietin-1 (Ang-1) and its receptor, Tie-2, another pro-angiogenesis signaling system. Finally, we found a tight correlation between WT1 expression and angiogenesis in primary Ewing sarcoma. Taken together, these findings support the hypothesis that WT1 is a key mediator of tumor angiogenesis in Ewing sarcoma.

## RESULTS

### Creation of transfected cell lines

**Figure 1:**
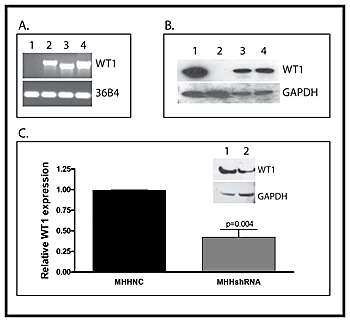
Creation of stably transfected cell lines A: RNA was isolated from SK-ES-1 cells transfected with an empty expression vector (Lane 1) or vectors directing expression of WT1A (Lane 2) or WT1D (Lane 3) The WT1-expressing cell line MHH-ES was used as a positive control (Lane 4). RNA was analyzed by RT-PCR for expression of WT1 using primers that span exon 5. The ribosomal RNA 36B4 was used as a loading control. B: Total protein was isolated from SKNC, SKWT1A and SKWT1D cell lines, as well as from MHH-ES cells, and Western blotting was performed with an antibody against WT1 (top panel) or GAPDH as a loading control (bottom panel). Lane 1: MHH-ES; Lane 2: SKNC; Lane 3: SKWT1A; Lane 4: SKWT1D. C: The WT1-expressing Ewing sarcoma cell line MHH-ES was transfected with either a WT1-specific shRNA or a scrambled control. RNA was isolated from the indicated cell lines and relative expression of WT1 mRNA was determined by quantitative RT-PCR. The signal obtained from the MHHNC cell line was arbitrarily assigned a value of 1.0, and signals were compared to this using the ΔΔCt method. Inset: Total protein was isolated from MHHNC (Lane 1) and MHHshRNA (Lane 2) cells, and western blotting was performed with an antibody against either WT1 (top panel) or GAPDH (lower panel) as a loading control.

WT1-null SK-ES-1 cells were transfected with an expression vector containing the cDNA for either WT1A or WT1D under the control of the CMV immediate early promoter, and stably transfected cells were selected for G418 resistance. SK-ES-1 cells transfected with the empty vector, referred to as SKNC cells, were used as a negative control. MHH-ES cells, which express all of the WT1 isoforms, were transfected with an expression vector containing a WT1-specific shRNA or a “scramble” negative control RNA under the control of the same CMV immediate early promoter, and stably transfected cells selected for G418 resistance. Successful expression of WT1 in the SK-ES-1 cells was confirmed by both RT-PCR and western blotting (Figure 1A and B). Successful suppression of WT1 in the MHH-ES cells was also confirmed by both qPCR and western blotting (Figure 1C). WT1 mRNA levels were reduced by 58.7 ± 9.33% in MHHshRNA cells (MHH-ES cells stably expressing WT1 shRNA) compared with MHHNC cells (MHH-ES cells transfected with the negative control RNA), and a similar reduction is also seen by western blotting.

### WT1 functions as a potent inducer of angiogenesis

**Figure 2:**
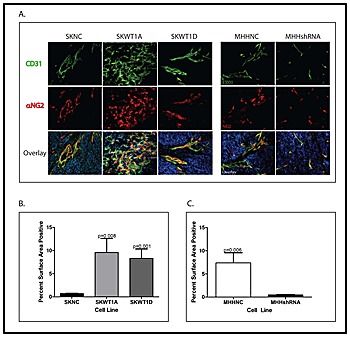
Effect of WT1 on tumor angiogenesis A: Mice xenografted with the indicated cell line were perfused with 4% paraformaldehyde, the tumors fixed, and paraffin-embedded slices stained with antibody against CD31 (green) and α-NG2 (red). Nuclei are counterstained with DAPI (blue). B: Total CD31 staining was quantified in SKNC, SKWT1A and SKWT1D xenografts from 10 fields from each group (10x objective), by position pixel algorithm using ImageJ software (NIH). Significance of the differences in CD31 staining were determined using Student's t test, and the p values are indicated. C: Total CD31 staining was quantified in MHHNC and MHHshRNA xenografts from 10 fields from each group (10x objective), by position pixel algorithm using ImageJ software (NIH). Differences in CD31 staining were evaluated using Student's t test, and the p values are indicated. All images are 200x.

To investigate the potential role of WT1 in tumor angiogenesis, stably transfected tumor cells were implanted subcutaneously into the flanks of NOD/SCID/IL-2Rγ null (NSG) mice. Tumors were harvested and vascularity was evaluated by immunohistochemistry using antibodies against the endothelial cell marker CD31 and the pericyte marker α-NG2. In comparing tumors arising from SK-ES-1 cells, there was substantially more staining with CD31 in WT1-expressing tumors compared with control (Figure 2A). Quantification of the total CD31-positive area in representative tumors showed an 8- to 9-fold increase in the WT1-expressing tumors (Figure 2B). In sections of tumors from the SKWT1A cells, 9.6± 2.8% of the surface area was stained for CD31, and in sections from the SKWT1D tumors, 8.3±2.0% of the surface area was stained for CD31. This compares with sections from the SKNC tumors, which had only 0.70±0.09% surface area CD31 positive. There were also profound morphologic differences in the vasculature of control tumors compared with tumors expressing WT1A and WT1D. Vessels in tumors arising from SKWT1A cells are slender, tortuous, and highly branched (Figure 2A) whereas those in tumors arising from SKWT1D cells are wide and long, with few branches, but readily apparent vascular sprouts and filopodial extensions (Figure 2A). We also evaluated angiogenesis in tumors arising from MHH-ES cells. Tumor vessel formation was suppressed in MHHshRNA tumors compared with MHHNC tumors (Figure 2A). Silencing of WT1 resulted in significantly less CD31-positive area in MHHshRNA tumors (0.48±0.075%) compared with MHHNC tumors (7.4±2.2%; p=0.006; Figure 2C). Interestingly, there were no morphologic differences in the vessels seen in tumors arising from MHHshRNA cells compared with tumors arising from MHHNC cells. Thus, in these Ewing sarcoma xenografts, modulating WT1 expression has a significant effect on the extent of tumor vasculature, and in conditions that alter the relative expression of isoforms, vascular morphology is also affected.

### WT1 modulates VEGF splicing in Ewing sarcoma cell lines

**Figure 3:**
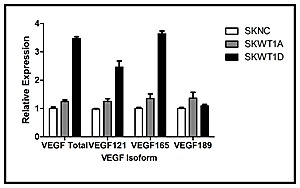
Effect of WT1 on VEGF isoform expression Total RNA was isolated from the indicated cell lines and analyzed for VEGF isoform expression using TaqMan RT-PCR. The signal obtained from the SKNC cell line was arbitrarily assigned a value of 1.0, and signals were compared to this using the ΔΔCt method. Bars indicate relative mRNA expression and error bars are the standard error of the mean of triplicate samples. This experiment was performed 3 times with similar results.

One of the most striking differences between the vessels seen in Ewing sarcoma xenografts that express only a single WT1 isoform is their morphology. Vessels in tumors derived from SKWT1A cells are slender and highly branched, whereas vessels in tumors derived from SKWT1D cells are wider, with less branching (Figure 2A). VEGF, the major factor regulating tumor vascularity, is subject to alternate splicing, resulting in expression of a variety of splice variants which can induce different vascular morphologies [21]. We therefore investigated the effect of WT1 isoforms on the expression of both total VEGF and of different VEGF isoforms in SK-ES-1 cells. RNA was isolated from SKNC, SKWT1A, and SKWT1D cells, and expression of total VEGF, VEGF121, VEGF165, and VEGF189 was evaluated using RT-PCR. Consistent with our previous report, the WT1A isoform induced a small, but statistically significant increase in VEGF expression (a 24% increase, p=0.035, Figure 3), while the WT1D isoform induced a substantial increase in total VEGF (a 3.5-fold increase, p<0.0001, Figure 3). In the SKWT1A cells, each VEGF isoform was modestly increased compared with SKNC cells, and all to the same degree (24-37%). In contrast, VEGF121 and VEGF165 were preferentially upregulated in SKWT1D cells (2.5- and 3.6-fold, respectively, p<0.0001 for each comparison), while VEGF189 expression was identical to control (Figure 3). Thus, expression of WT1D in SK-ES-1 cells can alter not only the amount of VEGF but also the splice variants that are expressed, and this may account for the distinctive morphology of vessels growing in these xenografts.

### WT1 modulates the expression of multiple genes that influence angiogenesis

**Figure 4:**
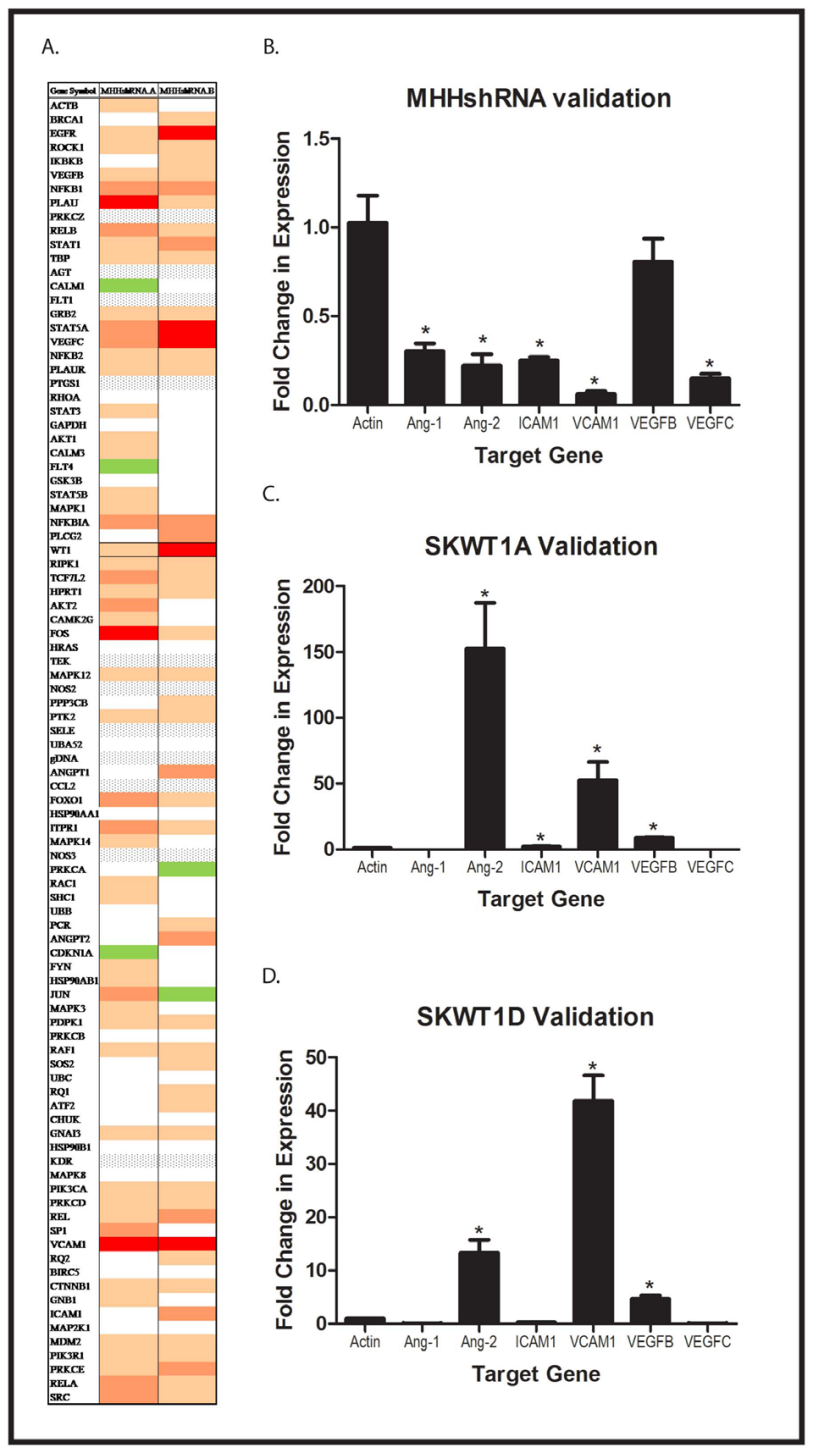
Global Effect of WT1 on Angiogenic Molecules A: Total RNA was isolated from 2 independently derived MHH-ES cell lines expressing a WT1 shRNA and from a MHH-ES cell line transfected with a scramble control. RNA was reverse transcribed, and the resulting cDNA analyzed using an angiogenesis PCR array as described. A heatmap of the results is presented. Red rectangles represent genes decreased by >75%, and varying shades of tan/orange represent less profound decreases. White indicates no difference, grey indicates no expression, and green represents > 1.3x increased expression. An independent RNA prep from (A) MHH shRNA and MHHNC, (B) SKWT1A and SKNC, and (C) SKWT1D and SKNC cells was reverse transcribed, and expression of each of the indicated target genes was assessed by qRT-PCR. Student's t test was used to assess differences for statistical significance, and the * indicates a p value < 0.05

**Figure 5:**
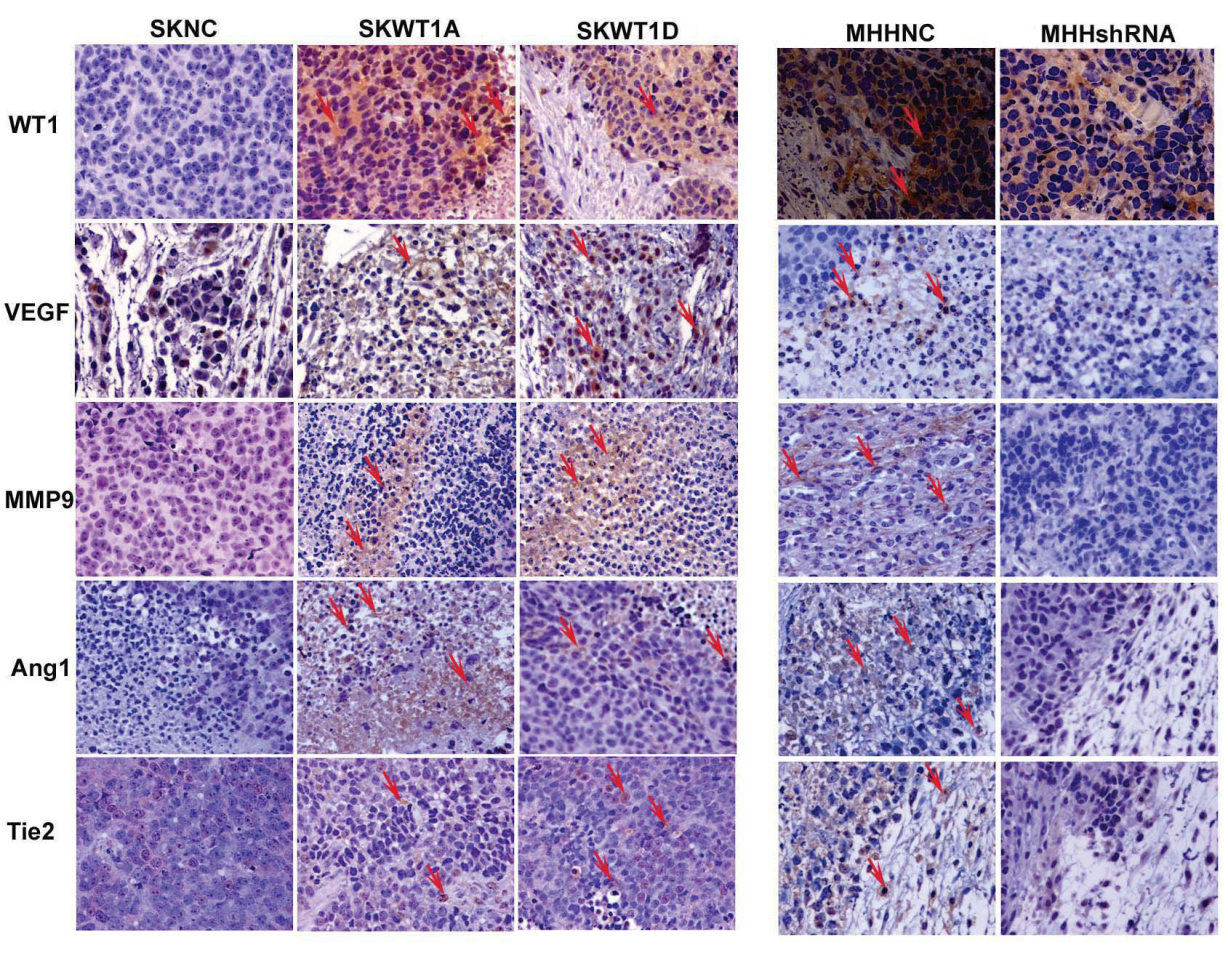
WT1 enhances expression of pro-angiogenic molecules Immunohistochemical analysis of tumors derived from the indicated xenografts. Serial sections of formalin- fixed, paraffin-embedded tumors were stained with the following antibodies: a mouse monoclonal WT1, a rabbit polyclonal VEGF, a rabbit polyclonal MMP9, a goat polyclonal Ang-1, and a goat monoclonal Tie-2. Signals were developed with DAB chromogen (brown) and counterstained with hematoxylin. Positive staining is shown with arrows. All images are 200x.

Our immunohistochemistry studies demonstrate that WT1 expression modulates tumor angiogenesis in Ewing sarcoma xenografts. Although we and others have shown that WT1 upregulates VEGF, a critical mediator of blood vessel growth, VEGF is not the only important pro-angiogenic signaling molecule. To determine whether other genes that influence angiogenesis are also regulated by WT1, we employed an angiogenesis PCR array. RNA was isolated from 2 independent MHH-ES clones with WT1 silenced by shRNA and from MHHNC cells, reverse transcribed, and assayed by RT-PCR for expression of a panel of genes associated with angiogenesis. Of the 90 genes on the array, 18 genes were decreased in expression by 50% or more upon silencing of WT1 (Figure 4A). We validated the suppression of 6 of these genes (ANGPT1, ANGPT2, ICAM1, VCAM1, VEGFB, and VEGFC) by performing RT-PCR on mRNA isolated from MHHshRNA and MHHNC cells using independent primer pairs (Figure 4B). In each case, the percent reduction in expression in the MHHshRNA cell lines matched that seen in the array. In a complementary experiment, we evaluated the expression of the same panel of genes in SKESNC cells and in SKWT1A and SKWT1D cells. Exogenous WT1A significantly upregulated expression of ANGPT2, ICAM1, VCAM1, and VEGFB (Figure 4C). Similarly, exogenous WT1D significantly upregulated ANGPT2, VCAM1,and VEGFB (but not VEGFC, Figure 4D). Thus, WT1 can either directly or indirectly regulate the expression of a number of pro-angiogenic target genes in ES cell lines. To determine whether, *in vivo*, WT1 can also modulate the expression of multiple angiogenic signaling molecules, we evaluated expression of angiopoietin-1 (Ang1) and its receptor, Tie2, as well as matrix metalloproteinase 9 (MMP9), which has previously been identified as a WT1 target gene in pulmonary epithelium [22]. Xenograft tumors generated from the cell lines described above (Figure 2) were harvested and evaluated by immunohistochemistry for expression of WT1, VEGF, MMP9, Ang1, and Tie2. SKWT1A and SKWT1D tumors have significantly more WT1, VEGF, MMP9, Ang1, and Tie2, as assessed by immunohistochemistry than did SKNC tumors (Figure 5). In contrast, MHHshRNA tumors contain significantly less of each of these factors when compared to the MHHNC tumors (Figure 5), further supporting our hypothesis that WT1 plays a central role in regulating tumor angiogenesis by modulating the expression of multiple angiogenesis-related signaling systems, both *in vitro* and *in vivo*.

### WT1 directly regulates the MMP9 promoter

**Figure 6:**
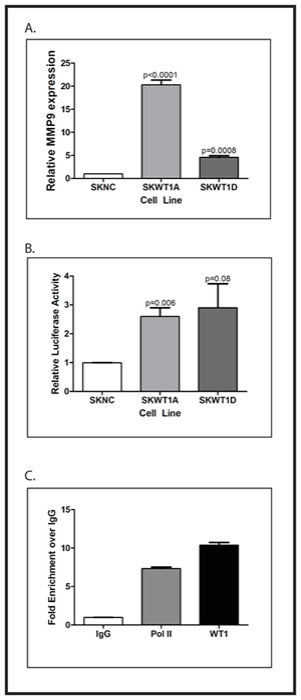
WT1 directly regulates MMP9 A. RNA was isolated from the indicated cell lines and relative expression of WT1 mRNA was determined by quantitative RT-PCR. The signal obtained from the SKNC cell line was arbitrarily assigned a value of 1.0, and signals were compared to this using the ΔΔCt method. Error bars represent standard error of the mean of experiments done in triplicate. Statistical significance was determined using Student's t test, with the indicated p values obtained. B: NIH3T3 cells were transfected with the MMP9 promoter-luciferase reporter construct and either the empty pCB6 expression vector or pCB6 containing the cDNA for the indicated WT1 isoform. Fold change is shown on Y-axis. Error bars represent standard error of the mean of experiments done in triplicate. Statistical significance was determined using Student's t test, with the indicated p values obtained. All experiments were repeated a minimum of three times. C: Chromatin from MHH-ES cells was immunoprecipitated with nonspecific IgG, or antibodies against RNA polymerase II (Pol II) or WT1. Co-precipitated DNA was analyzed by quantitative PCR using primers that flank the WT1 binding sites in the MMP9 promoter. The graph shows the fold enrichment in DNA immunoprecipitated by the indicated antibody compared with the control IgG.

MMP9 is a key regulator of tumor angiogenesis by virtue of its contribution to post-translational regulation of VEGF expression and bioavailability [23]. WT1 has been shown to suppress MMP9 expression in lung epithelial cells [22]. Because our WT1-expressing xenografts showed more MMP9 expression by immunohistochemistry, we were interested in whether MMP9 is also a direct WT1 target gene in Ewing sarcoma cells. To address this question, we measured MMP9 mRNA expression in SKNC, SKWT1A, and SKWT1D cells by quantitative RT-PCR. Both WT1-expressing cell lines expressed substantially more MMP9 than did control cells (Figure 6). NIH3T3 cells were co-transfected with a plasmid containing the cDNA for firefly luciferase under the control of the human MMP9 promoter and either an empty plasmid or a plasmid containing the cDNA for either WT1A or WT1D under the control of the constitutively active CMV immediate early promoter. WT1D caused a 3.5-fold stimulation of MMP9 promoter activity compared with control, while WT1A expression resulted in a 2.5-fold increase in MMP9 promoter activity (Figure 6). Both of these increases were statistically significant (p=0.006). Neither WT1A nor WT1D affected MMP2 promoter activity when compared to the control in similar experiments (data not shown).

To confirm that WT1 binds to the MMP9 promoter, ChIP assays were performed in the WT1-positive MHH-ES cell line. Chromatin was immunoprecipitated with a WT1 antibody, and the MMP9 promoter region (bases −142 to −84) containing a putative WT1 binding site was amplified by PCR. Using quantitative PCR, we were able to demonstrate an almost 10-fold enrichment of DNA containing the putative WT1 binding site in chromatin immunoprecipitated using antibodies against WT1 compared with a control IgG (Figure 6). These findings support the hypothesis that MMP9 is a direct WT1 target gene in Ewing sarcoma cell lines.

### WT1 expression modulates tumor growth *in vivo*

**Figure 7:**
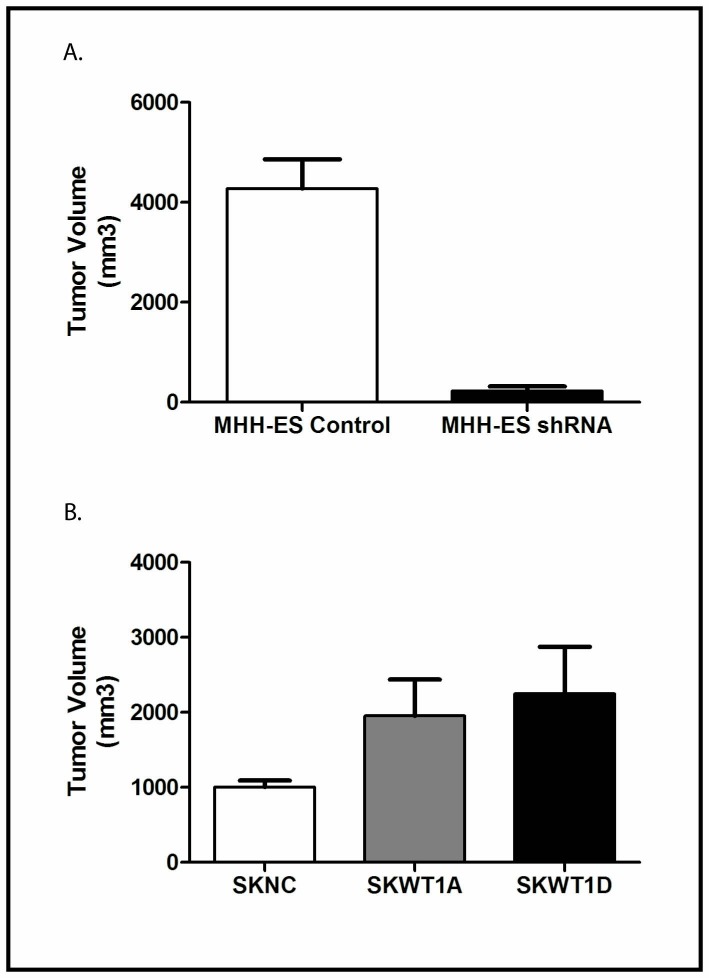
Effect of WT1 on Xenograft Growth A: The indicated cells were xenografted into NSG mice as described. Mice were euthanized when tumors in the largest group averaged 2,000 mm^3^. Data are the mean and SEM of cohorts of 5 mice. The difference is statistically significant with p < 0.0001. B: The indicated cells were xenografted into NSG mice as described. Mice were euthanized when tumors in the largest group averaged 2,000 mm^3^. Data are the mean and SEM of cohorts of 5 mice. Differences between SKWT1A and control and between SKWT1D and control are statistically significant at a p value of 0.08

Having demonstrated that WT1 expression modulates angiogenesis in our Ewing sarcoma xenografts, we next investigated whether this translated into an effect on tumor growth. We inoculated 10^6^ MHHshRNA or MHHNC cells subcutaneously into the flanks of NSG mice and measured tumor size every 4 days. Tumor growth was substantially diminished by knockdown of WT1 expression. Latency to tumor growth was 16 days. When mice bearing the largest tumors required euthanasia (estimated tumor volume of 2,000 mm^3^), all mice were euthanized, and tumor volumes measured. After 26 days, tumors grown from control cells reached an average size of 4278.4±585.4 mm^3^. Stably transfected MHHshRNA tumors grew to 229.8±86.5 mm^3^, a 90% inhibition of growth, which is statistically significant (p< 0.0001; Figure 7A).

To complement these experiments, we investigated whether exogenous WT1 would affect tumor growth in SK-ES-1 cells. We inoculated 10^6^ SKNC, SKWT1A, or SKWT1D cells subcutaneously into the flanks of NSG mice, and tumor size was measured twice a week. Latency to tumor formation was approximately 21 days. When mice bearing the largest tumors required euthanasia (estimated tumor volume of 2,000 mm^3^), all mice were euthanized, and tumor volumes measured. Control tumors grew to an average of 1004±88.6 mm^3^, while tumors from SKWT1A cells reached an average of 1956±483 mm^3^ and tumors from SKWT1D cells were an average of 2248±625 mm^3^ (Figure 7B). Although these differences did not quite reach statistical significance (p=0.08), when viewed in the context of the results with MHHshRNA tumors, these results confirm that WT1 expression modulates *in vivo* tumor growth of Ewing sarcoma xenografts.

### Co-expression of WT1, VEGF, MMP9 in Primary Ewing Sarcoma

**Figure 8:**
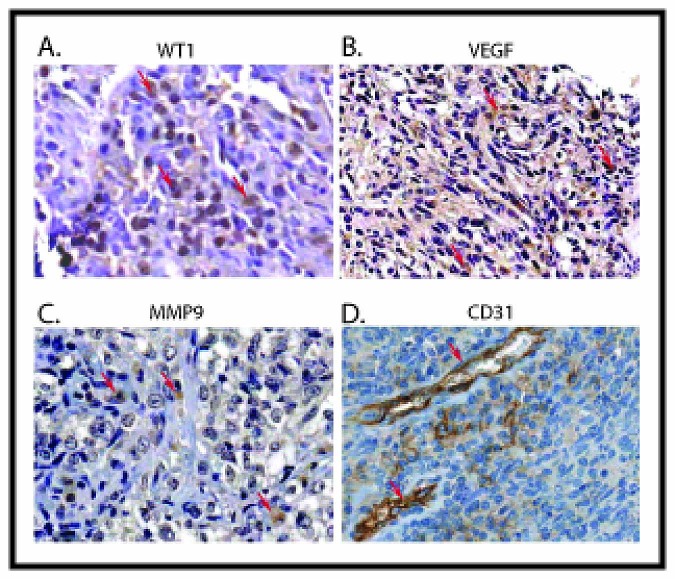
Correlation between the expression of WT1, VEGF, MMP9 and CD31 in Ewing sarcoma Ewing sarcoma tumor samples were immunostained with antibody to WT1 (A), VEGF (B), MMP9 (C), and CD31 (D). Signals were developed with DAB chromogen (brown) and counterstained with hematoxylin. Example of strong staining of corresponding antibody is illustrated. Positive expression is shown with arrows. All images are 200x.

To investigate whether VEGF and MMP9 expression in primary Ewing sarcoma correlate with WT1 expression, we performed immunohistochemical analysis of 21 paraffin-embedded Ewing sarcoma samples. The majority (16 out of 21, 76.2%) of the Ewing sarcoma samples stained for WT1 protein (Table 1 and Figure 8). In most of the samples, WT1 was detected in the cytoplasm as well as in the nuclei of cancer cells. Cytoplasmic staining of WT1 has been previously reported in rhabdomyosarcoma samples [1]. Eleven (52.4%) out of 21 samples showed strong and five (23.8%) samples showed moderate WT1 staining. Similarly, 8 out of 20 evaluable samples (40%) showed strong and 10 (50%) showed moderate VEGF staining (Figure 8). Finally, 8 of 20 evaluable samples (40%) had strong staining for MMP9 (Figure 8), and 9 samples (45%) had moderate staining. We also used immunohistochemistry to evaluate the vascularity of these tumors, staining for the endothelial cell marker, CD31 (Figure 8).

**Table 1:** Immunohistochemical labeling results for 21 Ewing sarcoma tumor samples

Antibody	Strong	Moderate	Weak
			
WT1	11	5	5
VEGF	8	10	2
CD31	11	3	6
MMP9	8	9	3

Ewing sarcoma samples on a custom tissue microarray were evaluated for expression of WT1, VEGF, CD31, and MMP9 by immunohistochemistry and scored for intensity of expression.

Positive staining for VEGF, MMP9, and CD31 correlated with WT1 staining in these samples. The WT1-negative tumors all had low or moderate VEGF staining, and the tumors with the strongest WT1 staining all had moderate or strong VEGF staining (Table 2). The Spearman rank correlation coefficient for WT1 and VEGF was 0.778 (p=0.0001). WT1 and MMP9 staining were also correlated – none of the MMP9-weak tumors expressed WT1, and all of the tumors with strong WT1 staining had either moderate or strong MMP9 staining (Table 3). The Spearman rank correlation coefficient for these proteins was 0.429 (p=0.05). Finally, WT1 staining correlated with CD31 immunostaining. Three of the 5 WT1-weak tumors had weak CD31 straining, whereas 8 of the 13 tumors with strong WT1 staining had strong CD31 staining (Table 4). The Spearman rank correlation coefficient for WT1 and CD31 was 0.431 (p=0.05). Together, these data demonstrate that, as was seen in cell lines and xenografts, WT1, VEGF, CD31 and MMP9 expression levels in primary Ewing sarcoma tissues are correlated with each other, supportive of the hypothesis that WT1 expression positively regulates tumor angiogenesis by upregulating VEGF and MMP9 expression.

**Table 2:** Correlation between WT1 and VEGF expression

		VEGF		
		Weak	Moderate	Strong
WT1	Weak	2	2	0
	Moderate	0	5	0
	Strong	0	3	8

A comparison of WT1 and VEGF expression in the TMA samples described in Table 1. The Spearman rank correlation coefficient for WT1 and VEGF co-expression was 0.778 (p=0.0001).

**Table 3:** Correlation between WT1 and MMP9 expression

		MMP9		
				
		Weak	Moderate	Strong
WT1	Weak	3	1	1
	Moderate	0	3	2
	Strong	0	4	5

A comparison of WT1 and MMP9 co-expression in the TMA samples described in Table 1. The Spearman rank correlation coefficient for WT1 and MMP9 was 0.429 (p=0.05).

**Table 4:** Correlation between WT1 and CD31 expression

		CD31		
		Weak	Moderate	Strong
WT1	Weak	3	1	1
	Moderate	1	1	2
	Strong	2	1	8

Effect of WT1 on tumor vascularity, as assessed by CD31 immunohistochemistry, in the TMA samples described in Table 1. The Spearman rank correlation coefficient for WT1 and CD1 was 0.431 (p=0.05).

## DISCUSSION

In this work we tested the hypothesis that WT1 expression regulates tumor angiogenesis. In a Ewing sarcoma xenograft model, we found that tumors which express high levels of WT1 demonstrate increased angiogenesis, while suppression of WT1 dramatically diminishes angiogenesis. Previous work has demonstrated that WT1 regulates VEGF expression [10, 24], but this is the first demonstration in an animal model that this translates into an increase in tumor angiogenesis. We also demonstrated increased expression of Ang1 and Tie2 in WT1-overexpressing tumors, suggesting a more global role for WT1 in promoting angiogenesis. The concept of WT1 as a global regulator of angiogenesis is further supported by our demonstration MMP9 is a direct target of the transcriptional regulatory activity of WT1 and identification of a small panel of additional pro-angiogenic molecules upregulated by WT1 in Ewing sarcoma cell lines. Finally, WT1 expression was correlated with VEGF expression, MMP9 expression, and microvessel density (as reflected by CD31 expression) in samples of primary Ewing sarcoma.

Neoangiogenesis is essential for tumor growth, and in many types of cancer, angiogenic activity parallels tumor aggression. Although the major drive for new vessel growth is hypoxia, low oxygen tension is insufficient to explain the differences in angiogenic activity reported in different tumor types as well as the variability seen among individual tumors of the same histology, suggesting that other factors also regulate tumor angiogenesis. We believe that WT1 may be one such factor. There are several lines of evidence that support a role for WT1 in regulating tumor angiogenesis: 1) WT1 expression has been demonstrated in tumor endothelial cells in a wide variety of common tumors [9] as well as in tumors derived from endothelial cells, such as Kaposi sarcoma and angiosarcoma [25, 26]; 2) our work and work from other labs has demonstrated that WT1 directly regulates the expression of VEGF [10, 24], the major cytokine regulating blood vessel growth; 3) WT1 expression can be regulated by hypoxia [10], and we have shown that knocking down WT1 expression with shRNA diminishes the HIF-1-mediated response to hypoxia [10], suggesting a functional role for WT1 in the response to hypoxia. More recently, Vadasz et al. reported that WT1 expression correlates with angiogenesis in lymph nodes affected by Hodgkin lymphoma, though importantly, this group demonstrated expression in endothelial cells, not in tumor cells [27]. The work reported here strengthens the hypothesis that WT1 is an important regulator of tumor angiogenesis by demonstrating the direct correlation between WT1 expression and angiogenesis in Ewing sarcoma xenografts as well as in primary tumors.

WT1 clearly regulates the expression of VEGF, but angiogenic activity is also influenced by other proteins such as angiopoietins (Ang-1 and -2), which are ligands for the Tie2 receptor, a tyrosine kinase receptor predominantly expressed in vascular endothelial cells. Ang1 binding to Tie2 induces multiple activities related to angiogenesis, such as endothelial cell migration, tube formation, sprouting, and survival [28, 29]. Upregulation of Ang1 has been reported in many malignancies including sarcomas [30-32]. Increased Tie2 expression also correlates with increasing tumor growth in many solid tumors [31, 33, 34]. Interestingly, in our tumor model we see increased expression of Ang1 and Tie2, in addition to VEGF, strengthening the hypothesis that WT1 is a more global regulator of angiogenesis. Further support for this hypothesis comes from our angiogenesis array experiments, which demonstrate that suppression of WT1 in Ewing sarcoma cells reduces expression of a significant number of pro-angiogenic genes. It remains to be determined whether WT1 directly or indirectly regulates Ang1, Tie2, or any of these other genes.

We also found that MMP9, a protease that plays a key role in the processing of a number of pro-angiogenic proteins, including VEGF, is a WT1 target gene. MMP9 is over expressed in many human cancers, and plays a critical role in tumor cell invasion, tumor growth, and angiogenesis by proteolytic degradation of extracellular components [35, 36]. Interestingly, MMP9 has been identified as a direct WT1 target genes in normal lung epithelial cells, but in those cells, MMP9 is downregulated by WT1 [22]. Our work supports the identification of MMP9 as a direct WT1 target gene, but suggests that the effect of WT1 on MMP9 expression is either tissue-specific or differs in normal cells and malignant cells, something to be investigated in the future.

Another interesting finding was the effect of different WT1 isoforms on vascular morphology. Vessels in tumors expressing exclusively WT1A are slender, tortuous, and highly branched, whereas those found in tumors expressing exclusively WT1D are wide and long, with few branches, but readily apparent vascular sprouts and filopodial extensions. Naturally occurring tumors express all 4 major WT1 isoforms simultaneously (albeit in varying ratios) masking these isoform-specific differences. Alternative splicing of the VEGF mRNA results in the expression of multiple different protein isoforms with distinct biological properties [37, 38]. Although expression of only a single isoform is sufficient to support vessel formation, the properties of vascular networks differ greatly depending on which isoform predominates during their growth [39]. Different VEGF isoforms can induce different vascular morphology [21, 40, 41], and we found that isoforms of WT1 differentially upregulate VEGF isoforms, providing an explanation for our observation that different WT1 isoforms induce vessels with distinct morphologies. Although the work of Amin et al. previously showed that WT1can influence VEGF splicing [20], this is an indirect effect based on changing expression of the splicing factor, SRPK1, and only affects the choice of alternate exons 8 (8a vs 8b). In contrast, our work shows a differential effect of individual WT1 isoforms on the internal, better characterized VEGF splice sites. Similar results were reported by Cunningham et al. who showed that loss of WT1 expression in hematopoietic progenitor cells results in an abnormal profile of VEGF isoforms [42]. We did not evaluate which exon 8 was included, but since both WT1A and WT1D increase angiogenesis, it is unlikely that either isoform alone increases the use of exon 8b (which is antiangiogenic) over exon 8a. Future work will investigate whether these splicing differences reflect a direct effect of WT1, or an indirect effect (via regulation of downstream splice factors). The differences between our results (which are similar to those of Cunningham et al.) and those of Amin et al. may have profound biological relevance. Perhaps WT1 has pro-differentiating (tumor suppressing) and anti-angiogenic activity when expressed in normal cells (such as hematopoietic stem cells and the developing kidney), but oncogenic and pro-angiogenic activity when expressed in pathologic conditions (such as in the context of abrupt hypoxia or neoplastic transformation). This will be an important focus of future work.

Originally identified as a tumor suppressor gene, WT1 can also act as an oncogene in some contexts [43]. This observation parallels the effect of WT1 on angiogenesis. Our work clearly shows that, in the context of Ewing sarcoma, WT1 is pro-angiogenic. This translates into increased tumor growth in xenografted cell lines. Suppression of WT1 in MHH-ES cells profoundly slows tumor growth, while exogenous WT1 increases the growth of SK-ES-1 xenografts. Although the increased growth did not reach statistical significance at the p < 0.05 level in SK-ES-1 cells, the trend toward increased growth was strong (p=0.08) and complements the effect of suppressing WT1 in MHH-ES cells. In our SK-ES-1 experiments, we expressed individual WT1 isoforms, but in cells that normally express WT1, multiple isoforms are expressed simultaneously. It is possible that the effect of WT1 on the growth of SK-ES-1 cells would be more profound if all WT1 isoforms were upregulated, and in normal ratios, rather than just one at a time. Regardless of the value arbitrarily set to assign significance, taken together, our findings with MHH-ES and SK-ES-1 cells support the hypothesis that WT1 is a pro-angiogenic factor in Ewing sarcoma, and this pro-angiogenic effect results in increased tumor growth.

The regulation of Ewing sarcoma angiogenesis is drawing increasing attention. Tilan et al. recently demonstrated that, in addition to upregulating VEGF, hypoxia also enhances Ewing sarcoma angiogenesis through induction of the neuropeptide Y (NPY) receptor Y2R in endothelial cells while also increasing the release of its ligand, NPY3-36, from Ewing sarcoma cells [44]. Zhou et al. also demonstrated that RE1-silencing transcription factor (REST), under the control of EWS-FLI1, controls Ewing sarcoma vascular morphology and tumor growth [45], though direct regulators of angiogenesis which might REST target genes were not identified. Our work implicates WT1 as a global regulator of Ewing sarcoma angiogenesis, and future work will clarify how NPY, REST, and WT1 interact.

In conclusion, our study provides support for a model wherein WT1 can influence tumor growth by regulating angiogenesis independent of tumor oxygenation. Our data identifies MMP9 as a novel WT1 target gene and demonstrates that WT1 expression directly regulates tumor growth through a global effect on angiogenesis. Further, our data support the notion that development of therapeutic strategies which target WT1 either by a small-molecule inhibitor or by an siRNA approach will provide effective treatment options for WT1-expressing tumors.

## MATERIALS AND METHODS

### Cells and Cell Culture

Ewing sarcoma cell lines MHH-ES and SK-ES-1 were cultured in RPMI 1640 supplemented with 10% fetal bovine serum (Gemini Bio-Products, West Sacramento, CA) and NIH3T3 cells were grown in DMEM (Invitrogen, Carlsbad, CA) supplemented with 10% fetal bovine serum (Invitrogen). Stably transfected cells were cultured in growth medium supplemented with 0.5 mg/ml G418 (Mediatech Inc, Manassas VA). All cells were maintained at 37 °C in 5% CO_2_.

### Stable Transfections

WT1A and WT1D expression plasmids have been described previously [10]. WT1 shRNA plasmids were purchased from SA Biosciences (Frederick, MD). SK-ES -1 and MHH-ES cells were seeded in six-well plates and grown to 80–90% confluence. Transfections were performed using Lipofectamine 2000 (Invitrogen) according to the manufacturer's instructions. Forty-eight hours after transfection, 1.0 mg/ml G418 (Gibco, USA) was added to cell cultures for selection. WT1 expression was confirmed in G418-resistant clones by real time quantitative RT-PCR and by western blot analysis. Stable transfectants were maintained in 0.5 mg/mg G418 and expanded for subsequent experiments.

### In Vivo Tumor Growth

All animal experiments were approved by the Johns Hopkins Animal Care and Use Committee. *In vivo* tumor formation was evaluated using NOD/SCID/IL-2 receptor γ null (NSG) mice. SKNC, SKWT1A, SKWT1D, MHHNC or MHHshRNA cells were implanted subcutaneously into the right flanks in a 1:1 mixture of Matrigel (BD Biosciences, San Jose, CA) and HBSS in a final volume of 200 μl in groups of 3-5 mice. Mice were sacrificed when tumors reached ~20 mm in diameter.

### Western Blot Analysis

Whole-cell lysates were prepared, and Western blot analysis was performed as described previously [10]. The primary antibody was anti-WT1 (Mouse monoclonal, 1:1000; Novus Biological, Littleton, CO) and anti- GAPDH (Rabbit polyclonal, 1:5000; Santa Cruz Biotechnology, Santa Cruz, CA) was used as a loading control.

### Reverse Transcription-Polymerase Chain Reaction (RT-PCR), qPCR, TaqMan RT-PCR, and Angiogenesis PCR Arrays

Total RNA was isolated from cultured cells and tumors using the RNeasy Mini Kit (QIAGEN Inc, Valencia, CA) according to manufacturer's instructions, followed by reverse transcription as previously described (Iscript Reverse Transcriptase, Bio-Rad, Hercules, CA). RT-PCR and qPCR were performed essentially as previously described [46]. WT1 oligonucleotides used for PCR amplification were described previously [10]. QPCR Primers for WT1 (PPH00254A), MMP9 (PPH00152E), and beta-2-microglobulin (PPH01094E) were obtained from SABiosciences (Frederick, MD). For TaqMan RT-PCR, probes specific to beta-2-microglobulin, total VEGFA and VEGF isoforms 121, 165 and 189 were obtained from Applied Biosystems (Grand Island, NY, USA). Quantification of gene expression was performed using a Bio-Rad CFX real-time PCR detection system with TaqMan chemistry in 96 well plates for 40 cycles of: 95°C for 15 s and 60°C for 1 minute followed by melt curve analysis. Angiogenesis arrays (Cat. # 100-25073) and primers specific to β-actin, ANGPT1, ANGPT2, ICAM1, VCAM1, VEGFB, and VEGFC, for array validation were purchased from Bio-Rad (Hercules, CA). The final list of primer assays used in the array is shown in the figure. Quantification of gene expression was performed using a Bio-Rad MyiQ single color real time PCR detection system with SYBR Green chemistry in 96-well plates for 1 cycle of 95°C, then 40 cycles of 95 °C for 5 s and 60 °C for 30 s, followed by melting curve analysis. For all RT-PCR methods, the mean threshold cycle (C_t_) of the triplicate samples was determined and then the C_t_ for each sample was corrected against the C_t_ level ofbeta-2-microglobulin or actin as indicated. Quantification of gene expression was performed by calculating ΔΔC_t_ where ΔΔC_t_ = (C_tsample_ – C_tcontrol_)_control_ – (C_tsample_ – C_tcontrol_)_treated_. The fold change in gene expression between two samples was then determined by calculating 2^−ΔΔCt^

### Immunohistochemistry

For evaluation of tumor angiogenesis in xenografts, at the time of sacrifice, mice were anesthetized with ketamine (100 mg/kg; i.p.) plus xylazine (10 mg/kg; i.p.) and perfused with 1% paraformaldehyde (Sigma-Aldrich) at 2 mL/min using cardiac puncture of the left ventricle. After perfusion with fixative, tissue was dissected and immersed in 1% paraformaldehyde for 2 hours followed by immersion in 30% sucrose (Sigma-Aldrich) for 48 hours. Tumors were then embedded in tissue-freezing media [ornithine carbamyl transferase (OCT); Tissue-Tek, Sakura Finetek, Torrance, CA, USA]. OCT blocks were sectioned (6 μm) and slides were immersed in 1% bovine serum albumin (Sigma-Aldrich) in PBS for 30 minutes. Sections were incubated overnight with the primary antibody, anti-NG2 Chondroitin Sulfate Proteoglycan (1:200, AB5320, Millipore) or anti-CD31 (1:50, 550274, BD Biosciences). Slides were washed with PBS and sections were incubated with the secondary antibody, fluorescein isothiocyanate-conjugated anti-rabbit Ig (1:400, 554020, BD Biosciences) or Cy3-conjugated anti-rat IgG (1:400, A10522, Invitrogen). Sections were counterstained with DAPI and mounted with Vectashield (Vector Laboratories). Sections were visualized using microscopy (Nikon E600), and photographed with a digital camera (Nikon DXM1200F).

Paraffin-embedded primary Ewing sarcoma tissue samples were deparaffinized in xylene, hydrated by a graded series of ethanol washes, and rinsed in 1x PBS. Antigens were retrieved by boiling samples for 10-15 minutes in citrate buffer pH6 (Invitrogen). Endogenous peroxidase was quenched using 3% hydrogen peroxide (Sigma, St. Louis, MO). Nonspecific binding sites were blocked using 1 mL PBS containing 5% goat serum and 1% bovine serum albumin (BSA). The sections were incubated overnight at 4 °C in a humidor with monoclonal antibody to WT1 (Mouse monoclonal,1:100; DAKO, clone 6F-H2), VEGF (1:100, Santa Cruz Biotechnology), CD31 (ab-2864, Abcam, Cambridge, MA), MMP9 (1:75, Cell Signaling Technologies), Ang-1 (1:50, R&D Systems, Minneapolis, MN), or Tie-2 (1:50, R&D Systems) diluted with 1% goat serum, 0.2% BSA and 0.3% Triton X-100 in PBS (pH 7.4), followed by washing with PBS. Sections were then incubated with peroxidase-conjugated secondary antibody (Jackson Immunoresearch Laboratories) of appropriate specificity. 3, 3’ -diaminobenzidine (DAB, Pierce) was used as substrate for peroxidase and counterstaining was performed with modified Harris hematoxylin solution (Sigma). Sections were dehydrated by passage through graded alcohol concentrations and finally xylene. Cover slips were mounted using DPX (Sigma). Completed immunostaining was visualized using microscopy (Nikon E600), and photographed with a digital camera (Nikon DXM1200F; ACT-1 software).

### Luciferase Assay

NIH3T3 cells were co-transfected with various WT1 expression vectors and an MMP9 promoter-luciferase reporter construct, kindly provided by Dr. Boyd (The University of Texas M. D. Anderson Cancer Center, Houston, TX) [47, 48], which contains the human MMP9 promoter (bases −670 to +54) cloned into the pGL3basic vector. The putative WT1 binding site in the MMP9 promoter, identified by Marcet-Palacios, et al., is located within a 42bp sequence beginning at position −131 relative to the transcription start site [22]. Cells were harvested and lysed 48 h after transfection. Luciferase activities were determined using the dual luciferase assay system (Promega, Madison, WI) according to the manufacturer's specifications. A vector expressing Renilla luciferase under the control of the SV40 promoter was used as a control for transfection efficiency. Individual assays were normalized by internal Renilla luciferase activity. Experiments were performed in triplicate and repeated three times with similar results. Data are expressed as mean relative luciferase activity ± standard error of the mean.

### Chromatin Immunoprecipitation (ChIP) Assay

Chromatin immunoprecipitation (ChIP) was performed according to manufacturer's recommendations (Active Motif, Carlsbad, CA). In brief, 10^6^ MHH-ES cells were cross-linked with 1% formaldehyde for 10 min at 37 °C and washed three times with ice-cold PBS, followed by enzymatic shearing of the fixed chromatin per manufacturer's protocol. Next, sheared chromatin was incubated with 2 μg of either anti-WT1 (Novus Biotechnology), anti-RNA pol II, or a negative control IgG (Active Motif). Chromatin bound to the antibody was pulled down with magnetic Protein G-coated beads, washed and eluted. Following crosslink reversal, the purified DNA was subjected to PCR amplification using primers specific for the region containing the putative WT1 binding site in the MMP9 promoter, sense primer: 5’- CTGCGGGTCTGGGGTCTTGC -3; antisense primer: 5’- CGCTCCTGTGACCCCACCCC – 3’ [22]. PCR fragments were analyzed on 2% agarose 1x TAE gel containing ethidium bromide and the size (196 bp) was compared with a molecular weight marker. These results were further confirmed with quantitative PCR using the same sense and antisense primers.

### Statistical Analysis

All of the experiments were performed at least three times. The results are expressed as mean ± SEM. Statistical comparisons were made using ANOVA or an unpaired two-tailed Student *t* test using Prism v5.0 software (GraphPad Software, Inc, La Jolla, CA). A p value <0.05 was considered significant. Correlations between WT1 and VEGF, CD31, and MMP9 were evaluated using the Spearman rank test using Stata v11.2 software (StataCorp, College Station, TX).

## References

[1] Carpentieri DF, Nichols K, Chou PM, Matthews M, Pawel B, Huff D (2002). The expression of WT1 in the differentiation of rhabdomyosarcoma from other pediatric small round blue cell tumors. Mod Pathol.

[2] Inoue K, Ogawa H, Sonoda Y, Kimura T, Sakabe H, Oka Y, Miyake S, Tamaki H, Oji Y, Yamagami T, Tatekawa T, Soma T, Kishimoto T, Sugiyama H (1997). Aberrant overexpression of the Wilms tumor gene (WT1) in human leukemia. Blood.

[3] Loeb DM, Evron E, Patel CB, Sharma PM, Niranjan B, Buluwela L, Weitzman SA, Korz D, Sukumar S (2001). Wilms' tumor suppressor gene (WT1) is expressed in primary breast tumors despite tumor-specific promoter methylation. Cancer Res.

[4] Miwa H, Beran M, Saunders GF (1992). Expression of the Wilms' tumor gene (WT1) in human leukemias. Leukemia.

[5] Miyoshi Y, Ando A, Egawa C, Taguchi T, Tamaki Y, Tamaki H, Sugiyama H, Noguchi S (2002). High expression of Wilms' tumor suppressor gene predicts poor prognosis in breast cancer patients. Clin Cancer Res.

[6] Sotobori T, Ueda T, Oji Y, Naka N, Araki N, Myoui A, Sugiyama H, Yoshikawa H (2006). Prognostic significance of Wilms tumor gene (WT1) mRNA expression in soft tissue sarcoma. Cancer.

[7] Srivastava A, Fuchs B, Zhang K, Ruan M, Halder C, Mahlum E, Weber K, Bolander ME, Sarkar G (2006). High WT1 expression is associated with very poor survival of patients with osteogenic sarcoma metastasis. Clin Cancer Res.

[8] Wagner KD, Wagner N, Bondke A, Nafz B, Flemming B, Theres H, Scholz H (2002). The Wilms' tumor suppressor Wt1 is expressed in the coronary vasculature after myocardial infarction. Faseb J.

[9] Wagner N, Michiels JF, Schedl A, Wagner KD (2008). The Wilms' tumour suppressor WT1 is involved in endothelial cell proliferation and migration: expression in tumour vessels in vivo. Oncogene.

[10] McCarty G, Awad O, Loeb DM (2011). WT1 Protein Directly Regulates Expression of Vascular Endothelial Growth Factor and Is a Mediator of Tumor Response to Hypoxia. J Biol Chem.

[11] Haber DA, Sohn RL, Buckler AJ, Pelletier J, Call KM, Housman DE (1991). Alternative splicing and genomic structure of the Wilms tumor gene WT1. Proc Natl Acad Sci U S A.

[12] Bruening W, Pelletier J (1996). A non-AUG translational initiation event generates novel WT1 isoforms. J Biol Chem.

[13] Scharnhorst V, Dekker P, van der Eb AJ, Jochemsen AG (1999). Internal translation initiation generates novel WT1 protein isoforms with distinct biological properties. J Biol Chem.

[14] Nakagama H, Heinrich G, Pelletier J, Housman DE (1995). Sequence and structural requirements for high-affinity DNA binding by the WT1 gene product. Mol Cell Biol.

[15] Rauscher F, JF Morris, OE Tournay, DM Cook, and T Curran (1990). Binding of the Wilms' tumor locus zinc finger protein to the EGR-1 consensus sequence. Science.

[16] Wang ZY, Qiu QQ, Enger KT, Deuel TF (1993). A second transcriptionally active DNA-binding site for the Wilms tumor gene product, WT1. Proc Natl Acad Sci U S A.

[17] Drummond IA, Rupprecht HD, Rohwer-Nutter P, Lopez-Guisa JM, Madden SL, Rauscher FJ, Sukhatme VP (1994). DNA recognition by splicing variants of the Wilms' tumor suppressor, WT1. Mol Cell Biol.

[18] Reynolds PA, Smolen GA, Palmer RE, Sgroi D, Yajnik V, Gerald WL, Haber DA (2003). Identification of a DNA-binding site and transcriptional target for the EWS-WT1(+KTS) oncoprotein. Genes Dev.

[19] Morrison AA, Venables JP, Dellaire G, Ladomery MR (2006). The Wilms tumour suppressor protein WT1 (+KTS isoform) binds alpha-actinin 1 mRNA via its zinc-finger domain. Biochem Cell Biol.

[20] Amin EM, Oltean S, Hua J, Gammons MV, Hamdollah-Zadeh M, Welsh GI, Cheung MK, Ni L, Kase S, Rennel ES, Symonds KE, Nowak DG, Royer-Pokora B, Saleem MA, Hagiwara M, Schumacher VA (2011). WT1 mutants reveal SRPK1 to be a downstream angiogenesis target by altering VEGF splicing. Cancer Cell.

[21] Ruhrberg C, Gerhardt H, Golding M, Watson R, Ioannidou S, Fujisawa H, Betsholtz C, Shima DT (2002). Spatially restricted patterning cues provided by heparin-binding VEGF-A control blood vessel branching morphogenesis. Genes Dev.

[22] Marcet-Palacios M, Ulanova M, Duta F, Puttagunta L, Munoz S, Gibbings D, Radomski M, Cameron L, Mayers I, Befus AD (2007). The transcription factor Wilms tumor 1 regulates matrix metalloproteinase-9 through a nitric oxide-mediated pathway. J Immunol.

[23] Hawinkels LJ, Zuidwijk K, Verspaget HW, de Jonge-Muller ES, van Duijn W, Ferreira V, Fontijn RD, David G, Hommes DW, Lamers CB, Sier CF (2008). VEGF release by MMP-9 mediated heparan sulphate cleavage induces colorectal cancer angiogenesis. Eur J Cancer.

[24] Hanson J, Gorman J, Reese J, Fraizer G (2007). Regulation of vascular endothelial growth factor, VEGF, gene promoter by the tumor suppressor, WT1. Front Biosci.

[25] Al Dhaybi R, Powell J, McCuaig C, Kokta V (2010). Differentiation of vascular tumors from vascular malformations by expression of Wilms tumor 1 gene: evaluation of 126 cases. J Am Acad Dermatol.

[26] Timar J, Meszaros L, Orosz Z, Albini A, Raso E (2005). WT1 expression in angiogenic tumours of the skin. Histopathology.

[27] Vadasz Z, Shasha-Lavsky H, Nov Y, Bejar J, Lurie M, Tadmor T, Attias D (2013). Wilms' tumor gene 1: a possible new proangiogenic factor in Hodgkin lymphoma. Appl Immunohistochem Mol Morphol.

[28] Koblizek TI, Weiss C, Yancopoulos GD, Deutsch U, Risau W (1998). Angiopoietin-1 induces sprouting angiogenesis in vitro. Curr Biol.

[29] Papapetropoulos A, Garcia-Cardena G, Dengler TJ, Maisonpierre PC, Yancopoulos GD, Sessa WC (1999). Direct actions of angiopoietin-1 on human endothelium: evidence for network stabilization, cell survival, and interaction with other angiogenic growth factors. Lab Invest.

[30] Stratmann A, Risau W, Plate KH (1998). Cell type-specific expression of angiopoietin-1 and angiopoietin-2 suggests a role in glioblastoma angiogenesis. Am J Pathol.

[31] Takahama M, Tsutsumi M, Tsujiuchi T, Nezu K, Kushibe K, Taniguchi S, Kotake Y, Konishi Y (1999). Enhanced expression of Tie2, its ligand angiopoietin-1, vascular endothelial growth factor, and CD31 in human non-small cell lung carcinomas. Clin Cancer Res.

[32] van der Schaft DW, Hillen F, Pauwels P, Kirschmann DA, Castermans K, Egbrink MG, Tran MG, Sciot R, Hauben E, Hogendoorn PC, Delattre O, Maxwell PH, Hendrix MJ, Griffioen AW (2005). Tumor cell plasticity in Ewing sarcoma, an alternative circulatory system stimulated by hypoxia. Cancer Res.

[33] Caine GJ, Blann AD, Stonelake PS, Ryan P, Lip GY (2003). Plasma angiopoietin-1, angiopoietin-2 and Tie-2 in breast and prostate cancer: a comparison with VEGF and Flt-1. Eur J Clin Invest.

[34] Tanaka S, Sugimachi K, Yamashita Yi Y, Ohga T, Shirabe K, Shimada M, Wands JR (2002). Tie2 vascular endothelial receptor expression and function in hepatocellular carcinoma. Hepatology.

[35] Egeblad M, Werb Z (2002). New functions for the matrix metalloproteinases in cancer progression. Nat Rev Cancer.

[36] Tomanek RJ, Schatteman GC (2000). Angiogenesis: new insights and therapeutic potential. Anat Rec.

[37] Houck KA, Ferrara N, Winer J, Cachianes G, Li B, Leung DW (1991). The vascular endothelial growth factor family: identification of a fourth molecular species and characterization of alternative splicing of RNA. Mol Endocrinol.

[38] Tischer E, Mitchell R, Hartman T, Silva M, Gospodarowicz D, Fiddes JC, Abraham JA (1991). The human gene for vascular endothelial growth factor. Multiple protein forms are encoded through alternative exon splicing. J Biol Chem.

[39] Ruhrberg C (2003). Growing and shaping the vascular tree: multiple roles for VEGF. Bioessays.

[40] Carmeliet P, Ng YS, Nuyens D, Theilmeier G, Brusselmans K, Cornelissen I, Ehler E, Kakkar VV, Stalmans I, Mattot V, Perriard JC, Dewerchin M, Flameng W, Nagy A, Lupu F, Moons L (1999). Impaired myocardial angiogenesis and ischemic cardiomyopathy in mice lacking the vascular endothelial growth factor isoforms VEGF164 and VEGF188. Nat Med.

[41] Stalmans I, Ng YS, Rohan R, Fruttiger M, Bouche A, Yuce A, Fujisawa H, Hermans B, Shani M, Jansen S, Hicklin D, Anderson DJ, Gardiner T, Hammes HP, Moons L, Dewerchin M (2002). Arteriolar and venular patterning in retinas of mice selectively expressing VEGF isoforms. J Clin Invest.

[42] Cunningham TJ, Palumbo I, Grosso M, Slater N, Miles CG (2013). WT1 regulates murine hematopoiesis via maintenance of VEGF isoform ratio. Blood.

[43] Loeb DM, Sukumar S (2002). The role of WT1 in oncogenesis: tumor suppressor or oncogene?. Int J Hematol.

[44] Tilan JU, Lu C, Galli S, Izycka-Swieszewska E, Earnest JP, Shabbir A, Everhart LM, Wang S, Martin S, Horton M, Mahajan A, Christian D, O'Neill A, Wang H, Zhuang T, Czarnecka M (2013). Hypoxia shifts activity of neuropeptide Y in Ewing sarcoma from growth-inhibitory to growth-promoting effects. Oncotarget.

[45] Zhou Z, Yu L, Kleinerman ES (2014). EWS-FLI-1 regulates the neuronal repressor gene REST, which controls Ewing sarcoma growth and vascular morphology. Cancer.

[46] Awad O, Yustein JT, Shah P, Gul N, Katuri V, O'Neill A, Kong Y, Brown ML, Toretsky JA, Loeb DM (2010). High ALDH activity identifies chemotherapy-resistant Ewing's sarcoma stem cells that retain sensitivity to EWS-FLI1 inhibition. PLoS One.

[47] Gum R, Lengyel E, Juarez J, Chen JH, Sato H, Seiki M, Boyd D (1996). Stimulation of 92-kDa gelatinase B promoter activity by ras is mitogen-activated protein kinase kinase 1-independent and requires multiple transcription factor binding sites including closely spaced PEA3/ets and AP-1 sequences. J Biol Chem.

[48] Yan C, Wang H, Boyd DD (2001). KiSS-1 represses 92-kDa type IV collagenase expression by down-regulating NF-kappa B binding to the promoter as a consequence of Ikappa Balpha -induced block of p65/p50 nuclear translocation. J Biol Chem.

